# Clinical and Sociodemographic Characteristics Associated with Access to Early Intervention Programs for Infants with Prodromes of Autism

**DOI:** 10.3390/jcm15031044

**Published:** 2026-01-28

**Authors:** Natasha Chericoni, Eugenia Conti, Valeria Costanzo, Francesca Ieri, Ilaria Colombino, Giulia Guainai, Benedetta Riva, Fabio Apicella, Andrea Guzzetta, Sara Calderoni, Costanza Colombi

**Affiliations:** 1 Istituto di Ricovero e Cura a Carattere Scientifico (IRCCS) Stella Maris Foundation, 56128 Pisa, Italy; 2 Department of Clinical and Experimental Medicine, University of Pisa, 56126 Pisa, Italy

**Keywords:** autism screening, parental concern, risk factors, clinical factors, sociodemographic factors

## Abstract

**Background/Objectives:** Increasing scientific evidence supports the importance of early diagnosis of autism spectrum disorder (ASD), followed by timely intervention, in optimizing developmental trajectories. Despite these advances, achieving an early diagnosis remains challenging, and substantial delays in the diagnostic process continue to be reported worldwide. This study aimed to describe the clinical and sociodemographic characteristics associated with early referral to a telehealth parent-mediated intervention program for infants at high likelihood for ASD, under 18 months of age, with the broader goal of informing clinical services in the field of neurodevelopmental disorders. **Methods:** Infants were evaluated by a multidisciplinary team using standardized measures to assess autism risk, developmental functioning, adaptive behavior, and parental stress. Potential differences in age at access to the program were examined by comparing families who were referred before versus after 12 months of age. **Results:** Of the 78 families who expressed interest in the program, 69 consented and 60 met eligibility criteria (male/female ratio = 40/20; mean age = 10.0 months). Families were evenly distributed across Italy, and 66% of parents held a university degree. Self-referral accounted for 62% of cases. Higher parental concern was associated with earlier referral and children referred after 12 months of age showed significantly lower developmental and adaptive functioning scores. **Conclusions:** These findings support the feasibility of identifying prodromes of autism within the first year of life and highlight gaps in specialized services for infants at elevated likelihood in Italy. Maternal concern and self-referral drove early consultation, underscoring the need for improved pediatric training. Future studies should assess longitudinal population-based screening and the feasibility and long-term impact of timely interventions in routine care.

## 1. Introduction

Autism spectrum disorder (ASD) is a complex, early-onset neurodevelopmental condition that persists across the lifespan, affects brain development and behavior, and is characterized by difficulties in social communication, restricted and repetitive patterns of behavior or interests, and sensory processing differences that can substantially impact everyday functioning [1,2].

A growing body of evidence shows that an autism diagnosis before the age of three is associated with markedly improved developmental outcomes, particularly in social functioning [3]. These advantages are thought to stem from the heightened neuroplasticity and behavioral flexibility of early childhood, which may enhance responsiveness to targeted interventions [4,5]. Recent work further indicates that interventions initiated within the first two years of life, when early atypicalities emerge and brain development is rapidly evolving, may lead to even greater long-term gains [6,7,8,9]. Further supporting the critical importance of intervention timing, a recent randomized controlled trial (RCT) demonstrated that toddlers on the autism spectrum achieve significantly better outcomes when treatment is introduced at an earlier developmental stage (i.e., at 18 months rather than nine months later) [10].

Despite these advances, achieving an early diagnosis remains challenging, and substantial delays in the diagnostic process continue to be reported across clinical settings. In their large-scale systematic review and meta-analysis including over 120,000 individuals from 40 countries, Van ’t Hof et al. [11] reported a current global mean age at autism diagnosis of approximately 60 months, highlighting that—despite improvements in early detection strategies—the timing of diagnosis remains highly variable and, in many cases, still occurs well beyond early childhood. Importantly, subgroup analyses restricted to studies including only children aged 10 years or younger yielded a mean age at diagnosis of 43.18 months (95% CI: 39.79–46.57), with a wide range across samples. Consistent with these findings, data from the United States indicate that the median age at first ASD diagnosis is approximately 47 months, with considerable regional variation ranging from 36 months in California to 69.5 months in Laredo, Texas [12]. Recent evidence from the Autism Care Pathway in Europe study further substantiates the concern that early diagnosis of ASD remains difficult to achieve in several European countries [13]. Despite increasing awareness and expanded service availability, substantial delays persist at multiple points in the diagnostic trajectory. As a consequence, many children receive a diagnosis after the age of three and begin intervention even later, thereby missing the developmental window characterized by the highest levels of brain plasticity [14,15].

In Italy, diagnostic delays remain a significant concern. Approximately 15% of families report waiting more than one year between the initial expression of developmental concerns and the first screening appointment, and in nearly one quarter of cases an additional year or more elapses before a confirmed diagnosis is obtained [13]. Healthcare in Italy is publicly funded rather than insurance-based and is organized at both national and regional levels, ensuring universal coverage for children. Within this system, all children born in or residing in Italy are assigned a state-funded family pediatrician at birth or upon moving to the country, independently of family income. Pediatricians serve as the primary point of contact from birth and provide ongoing care throughout childhood. This structure places them in a pivotal position for the early recognition of atypical developmental trajectories and for initiating timely referral pathways to specialized services [16]. Access to specialized services may vary regionally, with differences in service availability and waiting times that can influence the timing of evaluations [17]. Overall, this structured pathway, combined with universal coverage, means that parental concerns are typically filtered through the pediatrician, underscoring their central role in early identification and referral. In a recent clinical study that retrospectively analyzed the records of children aged 18 to 71 months who were evaluated at the Child and Adolescent Neuropsychiatry Unit of an Italian pediatric hospital between 2016 and 2023 following pediatricians’ clinical suspicion of autism, the mean age at first ASD diagnosis was 43.71 ± 13.6 months [18]. Earlier diagnosis was predicted by lower cognitive or intellectual functioning and the age at which initial concerns were raised, whereas being a first-born child was associated with delayed diagnosis [18]. In general, prolonged diagnostic timelines within Italian health services are likely influenced by a combination of factors that warrant further examination, including delayed referral to specialist assessment, extended waiting times within health services, and the presence of mild, heterogeneous, or overlapping clinical features that may lead clinicians to issue provisional rather than definitive diagnoses.

Within this framework, delays in autism diagnosis have been attributed to factors operating at the child, family, and service levels [19,20]. In their examination of diagnostic pathways in China, Qu et al. [20] reported an average delay of approximately 13 months between the emergence of parental concerns and receipt of a formal diagnosis. Although many parents became aware of developmental differences when their children were between 1 and 2 years of age, initial contact with healthcare professionals often occurred later, typically between 2 and 3 years. Longer overall delays were associated with limited proactive responses from healthcare providers, including infrequent referrals to autism specialists, and nearly one quarter of children initially received only a provisional diagnosis. Importantly, the nature of parental concerns appeared to shape the timing of diagnosis. Concerns related to social–emotional functioning were associated with an earlier age at diagnosis, while language-related concerns prompted more rapid help-seeking. In contrast, motor development concerns were linked to longer delays before initial consultation, and initial concerns in the sensory domain predicted an older age at diagnosis, suggesting that these early signs may be less readily recognized as indicative of autism risk. Family-level characteristics also played a role, with higher maternal educational attainment associated with longer delays before first hospital access, possibly reflecting greater caution in pursuing an autism diagnosis, and single-parent families experiencing significantly longer intervals between initial healthcare contact and confirmed diagnosis, underscoring difficulties in navigating the healthcare system without partner support.

More broadly, evidence regarding the role of socio-demographic factors in early versus late autism diagnosis remains heterogeneous and sometimes contradictory across studies. For example, parental age and educational level were not significantly associated with the timing of ASD diagnosis in a cohort of Italian preschoolers [18]. Conversely, a Turkish study reported that higher paternal education and socioeconomic status were associated with earlier autism diagnosis [21], and studies from the United States have similarly identified earlier diagnosis among children from families with higher parental education and income [22,23]. In line with these mixed findings, a recent systematic review of 50 studies published between 1987 and 2019 found inconsistent associations between age at ASD diagnosis and socio-demographic variables, whereas clinical characteristics—such as greater social communication impairments and the presence of intellectual disability—were more consistently associated with earlier diagnosis [24]. Taken together, these findings suggest that socio-demographic factors influence diagnostic timing in context-dependent ways, highlighting the need for further research to clarify their interaction with clinical and healthcare system determinants.

Beyond factors influencing the timing of referral and diagnosis, a substantial body of research has investigated prenatal, perinatal, and familial risk factors associated with ASD, with the aim of identifying populations that may benefit from closer developmental surveillance from infancy. Epidemiological studies consistently report a higher prevalence of ASD in males than in females, with male-to-female ratios typically ranging between 3:1 and 4:1 across countries and age groups [12,25]. In addition to sex-related differences, prematurity and small size for gestational age (SGA) have been consistently associated with an increased likelihood of ASD, with emerging evidence suggesting sex-specific patterns in these associations [26]. Within this framework, a recent systematic review and meta-analysis reported that the association between male sex and ASD risk appears to be particularly robust among preterm children [27]. Pregnancy- and birth-related complications have also been linked to increased ASD likelihood, further highlighting the role of early biological vulnerability [28]. Evidence regarding assisted reproductive technologies (ART) remains mixed: while some studies and meta-analyses report no overall increased ASD risk after adjustment for parental and demographic factors [29,30,31], others suggest possible technique-specific associations, particularly for intracytoplasmic sperm injection procedures [32]. In addition, younger siblings of autistic children represent a well-established high-risk group, with approximately 20% later meeting diagnostic criteria for ASD. Familial recurrence risk is further influenced by infant sex and family aggregation patterns, being higher when the infant is male, the proband is female, and when multiple older siblings are autistic [33,34,35]. Collectively, this body of evidence highlights the value of careful developmental surveillance in infants with known risk factors, to facilitate earlier recognition of ASD-related developmental trajectories.

In line with the goal of identifying children at heightened likelihood for ASD as early as possible and developing interventions specifically tailored for very young children with socio-communicative vulnerabilities, a telehealth parent-mediated intervention program has been established over the past three years at IRCCS Fondazione Stella Maris, a tertiary-level referral hospital in Pisa, Italy, through two research projects focused on very early intervention (Project VIITA “Feasibility of an intervention for infants with prodromal symptoms of Autism Spectrum Disorder (ASD) in the early stages of life”—pilot study, and Project FIRRST “Fostering Infant Responsivity and Reciprocity—Support to Thrive-A Multisite Randomized Control Trial of a Preemptive Telehealth Intervention for Infants with Early Signs of Autism Spectrum Disorders”—RCT study).

With the overarching goal of informing clinical services in the neurodevelopmental field regarding the early identification of infants and toddlers with ASD-specific vulnerabilities, the primary aim of this study was to describe the clinical and sociodemographic characteristics of infants exhibiting early prodromes of autism who were enrolled in parent-mediated early intervention projects. As a secondary aim, given the substantial age-related variability in early childhood, we examined differences related to the timing of first access to services by comparing families whose children were referred before versus after 12 months of age. Based on previous literature linking greater symptom severity and intellectual impairment to earlier identification [18,24], we hypothesized that earlier referral would be associated with more pronounced clinical impairments, including lower developmental functioning and more evident autism-related vulnerabilities.

## 2. Materials and Methods

### 2.1. Ethical Clearance and Informed Consent

The study was carried out in accordance with the standards for good ethical practice of the Declaration of Helsinki. Approval for the pilot study (feasibility assessment of the intervention program) was obtained from the Pediatric Ethics Committee of the Meyer University Hospital, Florence, Italy, on 21 February 2023 (protocol number: VIITA23 No. 1, dated 17 January 2023). Approval for the randomized controlled trial (RCT; efficacy assessment of the intervention program) was subsequently granted on 20 May 2024 (protocol number: VIITA23; Substantial Amendment No. 1, dated 30 April 2024; ClinicalTrials.gov: NCT06817746, registered on 10 February 2025). Written informed consent was obtained from all children’s parents.

### 2.2. Procedure

**Figure 1:**
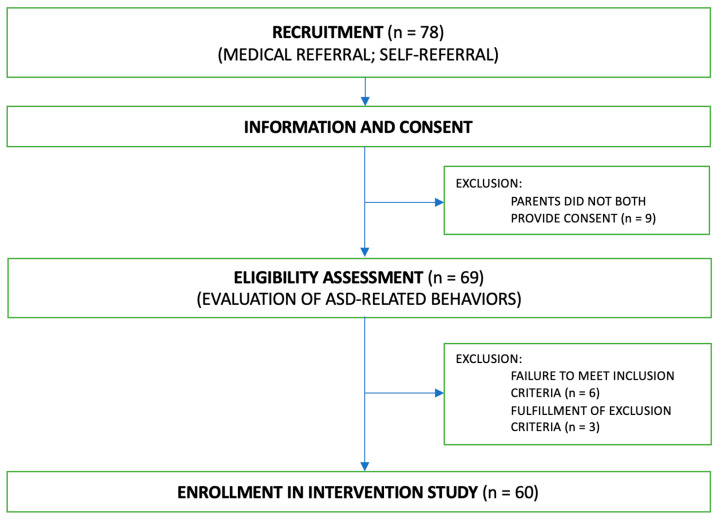
Flowchart on the Recruitment Process.

This study involved analyses of data collected from families who participated in a 6-month telehealth parent-mediated program developed at the IRCCS Fondazione Stella Maris (Pisa, Italy). Children were eligible to participate in the program if they were younger than 18 months at the time of recruitment, obtained a score within the risk range on the Social Attention and Communication Surveillance-Revised [36], and had at least one parent who spoke Italian sufficiently to communicate with the therapist; families were also required to have access to a webcam-equipped device with an internet connection to enable participation in the telehealth program. Exclusion criteria included the presence of a known genetic disorder, brain injury, or other relevant neurological or chronic medical conditions, as well as severe visual, auditory, and/or motor impairments. Recruitment was facilitated through dissemination using multiple outreach channels, including notifications to neuropsychiatric units across Italy, regional representatives of pediatricians, scientific meetings and conferences, social media, and a dedicated website (www.fiirrst.com). Referral to the program could be initiated either by medical professionals (pediatricians or other specialists) or directly by families, who could contact the research group via a dedicated email address advertised across all dissemination platforms or through the project website. A total of 78 families contacted the program to obtain additional information about the possibility of enrolling their infants. Of these, 69 families provided informed consent and were assessed for eligibility by a multidisciplinary team of autism specialists. Nine infants were excluded for not meeting inclusion criteria or for meeting exclusion criteria, resulting in a final sample of 60 participants (see Figure 1 for the Flowchart on the Recruitment Process).

### 2.3. Sample Characterization

#### 2.3.1. Infant Characteristics

Sixty infants at elevated likelihood for ASD were enrolled in this study (M/F = 40/20 age in months: M [SD] = 10.00 [3.49], range 2.5–17.5). All participants underwent a standardized protocol to evaluate autism-related symptoms (Social Attention and Communication Surveillance-Revised; SACS-R [36]), developmental functioning (Griffiths Scales of Child Development, Third Edition; Griffiths III [37,38]), adaptive behavior (Vineland Adaptive Behavior Scales, Second Edition; Vineland-II [39,40]) (See Table 1 for an overview of infants’ demographic and clinical characterization). Evaluations were carried out at IRCCS Fondazione Stella Maris, a tertiary-level referral hospital in Pisa, Italy, in a family-friendly room dedicated to the program. Assessments were conducted by a multidisciplinary team of experienced professionals (psychologists and child neuropsychiatrists) specialized in the early identification of ASD, who observed each infant over a 2 h period and reached a unanimous clinical judgment based on test results and consensus discussion. Following the assessment, families received a brief report summarizing test results and providing medical recommendations to be shared with their pediatrician. The telehealth intervention began the week following the assessment. At the time of enrollment, 53 participants (88%) were not receiving any other form of intervention, whereas 7 (12%) were undergoing psychomotor therapy or physiotherapy; of these, 2 were under 12 months of age and 5 were in their second year of life.

**Figure 2:**
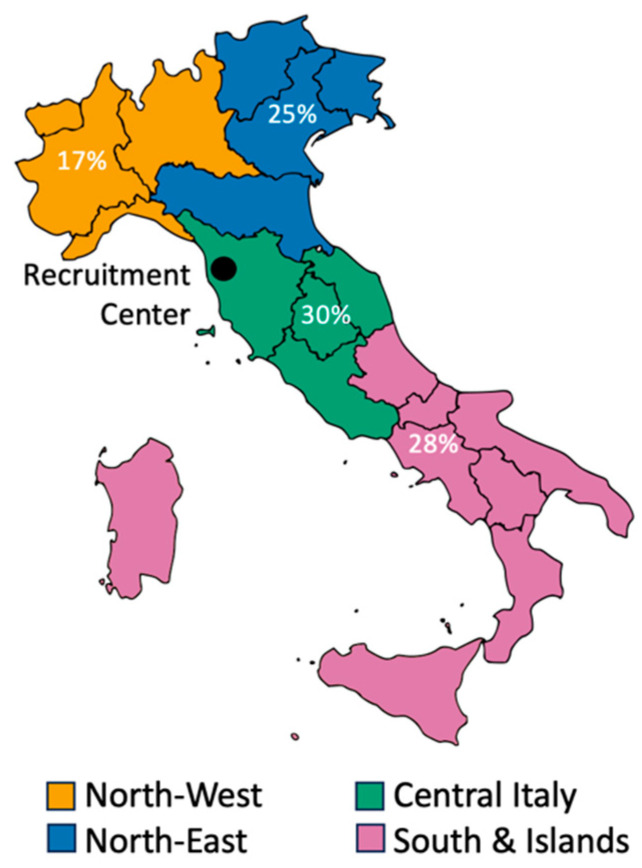
Geographical Distribution of Participants. Note. The location of the recruitment center (Pisa, Tuscany) is marked with a black circle.

Families were heterogeneously distributed across Italy (see Figure 2 for an overview of participants’ geographical distribution).

#### 2.3.2. Referral and Concern Categorization

Families could access the program either through medical referral or self-referral. Of the 60 infants enrolled in the intervention program, only 23 (38%) were referred by a medical professional, while most participants (37 infants, 62%) were self-referred by families, underscoring the prominent role of parental concern in initiating access to early intervention services.

Analysis of parental concerns at the time of first contact indicated that, regardless of referral pathway, parents were primarily concerned about behaviors commonly associated with ASD. These included reduced eye contact, diminished shared affect, heightened interest in non-social stimuli, and difficulties orienting to the caregiver’s face and voice, as well as the presence of mannerisms or repetitive behaviors. Overall, parents tended to express concerns mainly related to immaturities or atypicalities in the social-communication domain. Approximately half of the parents also reported concerns related to restricted or repetitive interests and behaviors, whereas families rarely contacted the program solely due to concerns in this latter domain. An overview of parental concerns, categorized according to DSM-5 criteria [1], is presented in Table 2.

#### 2.3.3. Parental Demographics and Stress Assessment

Mean parental age was mid thirties and level of education comprised completion of at least high school for the majority of parents (see Table 3 for an overview of parents’ demographic information).

**Figure 3:**
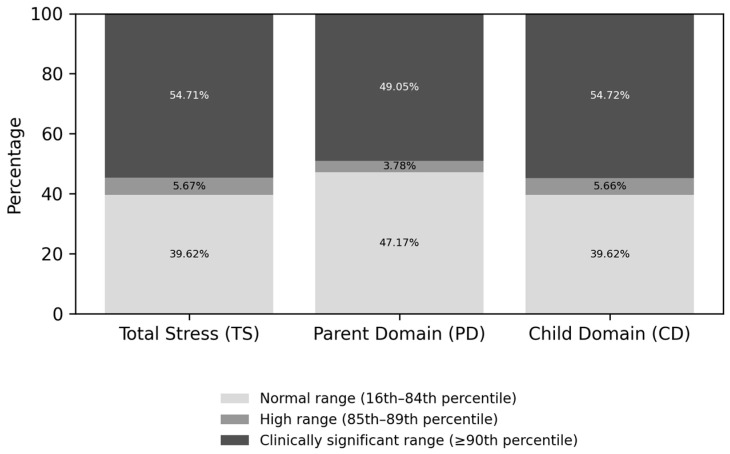
Percentage distribution of maternal stress levels (normal, high, and clinically significant), as assessed by the PSI-4.

Maternal stress levels, as measured by the Parenting Stress Index–4 (PSI-4 [41,42]), were in the clinically significant range for approximately 50% of parents (see Figure 3 for an overview of maternal stress levels at enrollment).

### 2.4. Materials and Design

#### 2.4.1. Social Attention and Communication Surveillance-Revised (SACS-R)

The SACS-R is an observational developmental surveillance instrument designed to detect early indicators of ASD in community settings [4,36,43]. It targets 12–15 age-specific social-communication behaviors assessed during routine early childhood surveillance. The SACS-R has shown robust psychometric properties across validation studies, high positive and negative predictive values (PPV: 82–83%; NPV: 98–99%), good sensitivity (77–82%), and excellent specificity (99–99.5%) [4,25]. In the present study, the SACS-R was employed to identify infants at high likelihood for ASD, who might benefit from a parent-mediated intervention supporting early social and communication development.

#### 2.4.2. Griffiths Scales of Child Development, Third Edition (Griffiths III)

The Griffiths III is a standardized measure of developmental functioning suitable for children from birth to 6 years of age and is commonly used in both clinical practice and research contexts [37,38]. It evaluates five domains of development: Foundations of Learning, Language and Communication, Eye–Hand Coordination, Personal–Social–Emotional Development, and Gross Motor Skills. Performance within each domain is reported using raw scores, age-equivalent scores, and standardized scores (M = 100, SD = 15), alongside a General Developmental Score that summarizes overall developmental level. The instrument has demonstrated robust reliability and validity and has been widely applied in studies of children with neurodevelopmental disorders, including autism spectrum disorder [44]. This measure was administered to assess the presence of developmental, language, and motor delays in children referred to the intervention program and to distinguish potential differences based on age at first access to the service.

#### 2.4.3. Vineland Adaptive Behavior Scales, Second Edition (Vineland-II)

The Vineland-II is a norm-referenced caregiver-report assessment used to measure adaptive functioning in daily life [39]. It examines adaptive behavior across four core domains: Communication, Daily Living Skills, Socialization, and Motor Skills. Administration is conducted through a semi-structured interview with caregivers, yielding standard scores, age-equivalent scores, and an Adaptive Behavior Composite derived from normative samples. The Vineland-II has well-established psychometric properties and is extensively used in both research and clinical settings involving children with neurodevelopmental disorders [39,40]. This measure was administered to assess adaptive behavior and examine differences by age at first access to the service.

#### 2.4.4. Parenting Stress Index—Fourth Edition (PSI-4)

The PSI-4 is a widely utilized questionnaire designed to assess levels of stress associated with parenting, particularly in families of children with developmental or clinical conditions [41,42]. The measure comprises 85 items organized into two primary domains. The Child Domain captures stress related to child characteristics, including mood, adaptability, distractibility, and parent–child reinforcement patterns, whereas the Parent Domain assesses stress associated with parental functioning, such as perceived competence, social support, responsiveness, and role restrictions. Percentile scores are used for interpretation, with values between the 16th and 84th percentiles considered typical, the 85th–89th percentiles indicating elevated stress, and scores at or above the 90th percentile reflecting clinically significant stress. This measure was administered to assess maternal stress at referral and examine associations with children’s social impairment and age at program access.

### 2.5. Data Analysis

Chi-square tests of independence were performed to examine the relationship between type of referral (self-referral vs. medical referral) and parental educational attainment (higher vs. lower), and the relationship between age at enrollment (<12 months vs. ≥12 months) and the presence of risk factors (at least one risk factor vs. no risk factors). Chi-square was chosen because all variables were categorical and observations were independent. For the comparison between age at enrollment and the presence of risk factors, one cell had an expected count <5; therefore, Fisher’s Exact Test was used to ensure accurate *p*-values.

Independent-samples *t*-tests were conducted to examine differences in infants’ clinical characteristics (Griffiths III developmental quotients and Vineland-II standard scores) by gender (female vs. male) and by age of referral (<12 months vs. ≥12 months). Most variables were approximately normally distributed, although a few showed deviations from normality (Shapiro–Wilk *p* < 0.05). Given the moderate sample size and the robustness of parametric tests to mild deviations from normality, parametric analyses were used for all variables. Non-parametric Mann–Whitney U tests were performed to assess whether SACS-R scores differed by gender or by age at referral. This test was selected because the SACS-R scores were not normally distributed, as indicated by skewness, kurtosis, and significant Shapiro–Wilk tests (*p* < 0.05). The Mann–Whitney U test does not assume normality and is appropriate for comparing two independent groups when the dependent variable is ordinal or non-normally distributed.

Spearman’s rank-order correlations were used to examine relationships between parental stress and age at enrollment, as well as between parental stress and infants’ clinical measures. PSI-4 percentiles for Total Stress, Parent Domain, and Child Domain showed moderate-to-strong skewness and significant Shapiro–Wilk values (*p* < 0.001), indicating non-normal distributions. The non-parametric Spearman method was therefore chosen, as it does not assume normality and is robust to skewed data.

## 3. Results

### 3.1. Association Between Parental Educational Attainment and Type of Referral

The Chi-square test indicated a significant association between parental educational attainment and type of referral (Table 4). Specifically, parents with higher educational attainment (university or doctoral degree) were more likely to self-refer, whereas parents with lower educational attainment (middle or high school diploma) were more likely to be referred by a specialist.

### 3.2. Association Between Presence of Risk Factors and Age at Enrollment

The Chi-square test of independence showed no significant association between the presence of risk factors and age at enrollment (Table 5). The distribution of risk factors was similar across the two age groups, with the presence of at least one risk factor being the most common condition in both age categories.

### 3.3. Comparison of Clinical Characteristics Between Male and Female Infants at Enrollment

The Mann–Whitney U test did not reveal statistically significant differences between female and male infants on the SACS-R measure of autism risk. Independent-samples *t*-tests revealed a trend-level effect for the Language and Communication domain on the Griffiths III scales, *t*(57) = −1.98, *p* = 0.052, with females showing slightly lower scores than males (M difference = −7.40, 95% CI [−14.88, 0.07]). No other significant gender differences were found across scores on the Griffiths III scales or on the Vineland-II domains.

### 3.4. Comparison of Clinical Characteristics Between Infants Younger and Older than 12 Months

**Figure 4:**
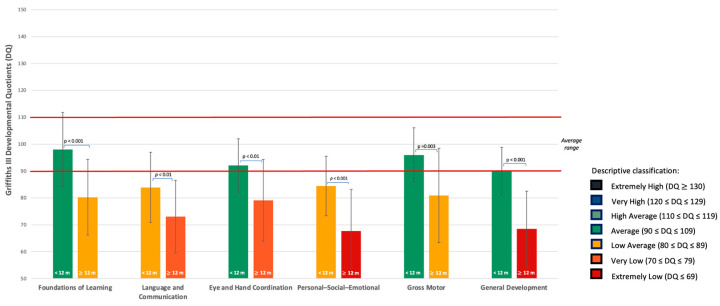
Griffiths III Developmental Profiles in Infants by Age at Enrollment. Group mean Developmental Quotients (DQ) are presented for infants enrolled before 12 months (<12 m) and at or after 12 months (≥12 m) across five developmental domains (Foundations of Learning, Language and Communication, Eye and Hand Coordination, Personal–Social–Emotional, Gross Motor), as well as the General Development Quotient. Error bars indicate standard deviations. Bar colors correspond to DQ descriptive ranges as indicated in the legend on the right. Statistically significant differences between groups are indicated (*p* < 0.05).

**Figure 5:**
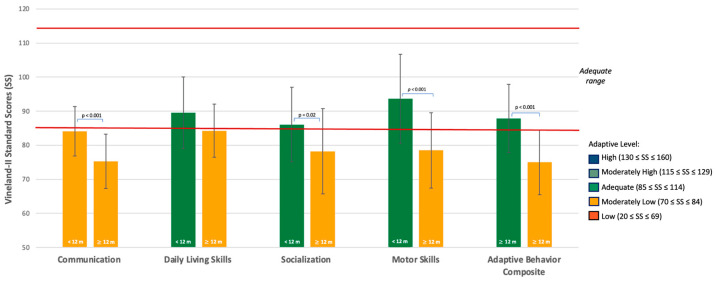
Vineland-II Adaptive Behavior Profiles in Infants by Age at Enrollment. Group mean Standard Scores (SS) are presented for infants enrolled before 12 months (<12 m) and at or after 12 months (≥12 m) across four adaptive domains (Communication, Daily Living Skills, Socialization, Motor Skills), as well as the Adaptive Behavior Composite. Error bars represent standard deviations. Bar colors correspond to adaptive levels, as shown in the legend. Statistically significant differences between groups are marked (*p* < 0.05).

The Mann–Whitney U test did not reveal statistically significant differences between infants referred before 12 months and those referred at or after 12 months on the SACS-R. Independent samples *t*-tests revealed significant differences between infants younger and older-referred infants. Younger-referred infants obtained higher scores on all Griffiths III scales (*p* < 0.01) and on most Vineland-II domains (*p* < 0.05), except for Daily Living Skills, where the difference did not reach statistical significance (*p* = 0.06) (See Table 6 and Figure 4 and Figure 5).

### 3.5. Correlations Between Parental Stress, Age at Enrollment, and Infants’ Clinical Measures

**Figure 6:**
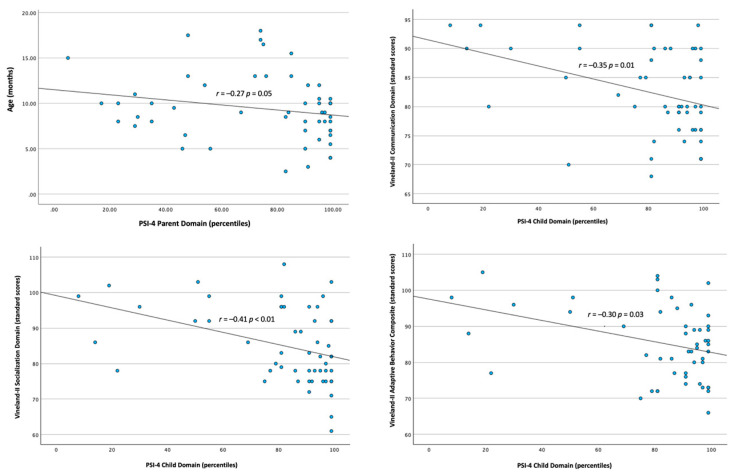
Correlation between Parents’ Level of Stress, Infants’ Age at Enrollment and Vineland-II Domains.

Spearman’s non parametric correlations showed a negative correlation (*r* = −0.27; *p* = 0.049) between maternal stress at the Parent Domain Scale of PSI-4 and infants’ age at enrollment, and between maternal stress at the Child Domain Scale and Scores at the Vineland II Communication (*r* = −0.35; *p* = 0.01), Socialization (*r* = −0.41; *p* = <0.01) and Composite scales (*r* = −0.30; *p* = 0.03) (see Figure 6). That is, higher maternal stress was associated with seeking help for their infants at a younger age. Moreover, higher maternal concern for their infants was associated with lower parent-reported communication and socialization scores on the Vineland-II interview.

## 4. Discussion

Despite our research institute being based in Central Italy, participants were evenly distributed across the country, representing families from the Northern, Southern, and Insular regions of Italy. This finding was partially unexpected as previous research had identified an uneven distribution of Healthcare Services for Autism services across the Italian territory, with fewer resources in the South and the Islands [17]. The high participation from families in Northern Italy may reflect the accessibility of our telehealth program, which can be delivered remotely, and may also underscore gaps in early identification and intervention services within the Italian healthcare system. Indeed, limited investment in specialist staff training within public healthcare services, coupled with the greater availability of diagnostic compared with intervention services [17], likely reduces the capacity to respond effectively to the rising number of early referrals for suspected autism and to meet families’ support needs. Although progress has been made in the early identification of autism-related behavioral markers, substantial delays in the diagnostic pathway remain common across clinical settings in Italy as well as worldwide [11,13].

Our early intervention projects were disseminated through general practitioners, social media, and a dedicated website (www.fiirrst.com) to maximize outreach. Notably, the majority of enrolled families self-referred (62%), with only 38% being referred by their pediatrician or another medical specialist. This pattern aligns with findings from Mendez et al. [13], who reported that in Europe parents or family members (70%) were usually the first to notice atypical development or behaviour in a child, while public health professionals raised such concerns in only 6% of cases.

The families enrolled in our early intervention projects generally had a high level of education, with 61% of mothers holding a higher education degree (university or doctoral) and 67% of families having at least one parent with a higher education degree. This pattern may reflect the tendency of parents with higher education to actively seek information and guidance from multiple sources, whereas those with lower educational attainment may rely more heavily on pediatricians. This interpretation aligns with previous literature indicating that higher parental education is associated with earlier ASD diagnosis [19,45].

During the anamnestic interview, it emerged that parents who self-referred expressed concerns similar to those reported by families referred by health professionals. In particular, parents appeared worried about both core ASD domains, including deficits in social communication and social interaction, as well as restricted and repetitive patterns of behavior and interests (DSM-5 Categories A and B), or, in some cases, solely about deficits in social communication and interaction (DSM-5 Category A). Only one family reported concern exclusively about repetitive behaviors (DSM-5 Category B), and only two families (both referred by health professionals) expressed no concerns. In line with previous investigations, parents appeared to recognize early vulnerabilities primarily in their infants’ social-communication development, including reduced eye contact, limited social responsiveness, inconsistent responses to their name, decreased social smiling, reduced imitation behaviors, diminished use of gestures and socially directed vocalizations, and increased attention to non-social stimuli [46,47,48,49]. Therefore, parental concerns were consistent with the focus of our intervention projects, which targeted immaturities and atypicalities in the socio-communicative domain. It is important to acknowledge, however, that, as reported in previous studies, parents’ initial concerns may not be limited to autism-specific characteristics but often include less ASD-specific difficulties, such as motor impairments, inattention and hyperactivity, cognitive and developmental delays, skill regression, tantrums or inappropriate behaviors, and eating or sleeping problems [50,51,52]. Consequently, pediatricians and other primary care professionals should carefully consider all parental concerns, as they may reflect more complex neurodevelopmental profiles.

In this cohort of infants referred for elevated likelihood of ASD, several established risk factors were highly prevalent, including prematurity (15%), small size for gestational age (SGA, 15%), pregnancy or birth-related complications (47%), assisted reproductive technologies (ART, 22%), and familial recurrence (22%). With respect to evidence indicating that the association between male sex and ASD risk is particularly robust among preterm children and those born SGA [26,27], our sample showed a partially divergent pattern: SGA was more frequent among males (17.5% vs. 10%), whereas prematurity was more common among females (20% vs. 12.5%). This finding is consistent with previous work demonstrating sex-specific differences in ASD risk as a function of gestational age, whereby males show elevated risk across all degrees of prematurity, while in females the association between earlier gestational age and ASD risk gradually attenuates as gestational age advances [53]. Together, these findings highlight the importance of careful monitoring of ASD risk in very early preterm females. Given the high global prevalence of preterm birth (approximately 11%) and survival rates exceeding 95% with modern neonatal care, even modest increases in ASD risk among preterm survivors may have substantial public health implications [54].

Pregnancy or birth-related complications were the most common risk factor in this cohort, affecting approximately half of the sample, with no sex differences. This finding aligns with previous evidence linking perinatal adversity to increased ASD likelihood [28].

ART was reported in approximately one fifth of the sample, with no sex differences. In line with the mixed evidence in the literature, this finding should be interpreted descriptively, without inferring a causal association between ART and ASD risk [29,30,31,32].

Approximately one fifth of infants were younger siblings of autistic children; 30% of females and 17.5% of males in the cohort had an affected older sibling. These findings underscore the importance of monitoring infants of both sexes in families with established ASD recurrence patterns [33,34,35].

Notably, the male-to-female ratio in this very young sample was 2:1, which is lower than the ratios typically reported in later childhood (3.4:1–4.4:1) [12,25]. Our lower male-to-female ratio may reflect improved recognition of early ASD signs in girls presenting a more typical ASD phenotype [55]. In contrast to reports at later ages [56,57,58,59], no significant sex differences emerged in our very young cohort across clinical and developmental measures, with the exception of a slight tendency for lower language and communication scores in females. This finding aligns with evidence indicating minimal sex differences in early ASD manifestations [60,61]. At this young age, camouflaging behaviors—known to contribute to under-recognition of ASD in females—are less likely to be present [62], which may partly explain the higher male predominance observed in school-aged samples [63,64,65]. Consistent with previous studies, our results support a male-to-female ratio of approximately 2:1 in infants at elevated likelihood for ASD [33,66,67], underscoring the importance of systematic developmental monitoring to improve early identification in females.

Overall, 78% of infants in our sample presented at least one risk factor, while 22% had no known risk factors. Parents tended to contact our service before their child’s first birthday regardless of the presence of risk factors, thereby reducing the likelihood of a referral bias linked to risk conditions. The high proportion of infants referred during their first year of life (70% of the cohort), compared with only 30% referred in the second year, underscores the importance of specialized services for early detection of autism-related developmental concerns to enable timely support and intervention. Notably, families often reported having contacted multiple healthcare professionals prior to enrollment, yet their concerns were frequently minimized or attributed to typical developmental variability, with reassurance that it was too early to evaluate for autism.

At baseline, all enrolled infants met at least three of the five key items on the SACS-R, indicating an elevated risk for autism. When examining potential age-related differences in clinical profiles, infants referred before 12 months of age showed mean Developmental Quotient (DQ) scores in the Low Average range on the Language and Communication and the Personal-Social-Emotional subscales of the Griffiths III, whereas the mean General DQ score remained within the Average range. On the Vineland-II Interview, their Communication domain fell in the Moderately Low range. In contrast, infants referred after 12 months of age exhibited significantly lower mean scores on all Griffiths III subscales and on the General Quotient, as well as on most Vineland-II domains (Communication, Socialization, Motor Skills, and Composite Scale) compared with those referred earlier. Specifically, infants in their second year of life had DQ scores in the Very Low range for the Language and Communication subscale and in the Extremely Low range for the Personal-Social-Emotional subscale. Other subscales were also impaired, including Foundations of Learning (Low Average), Eye and Hand Coordination (Very Low), Gross Motor (Low Average) and General Quotient (Extremely Low). On the Vineland-II scales, adaptive behavior was Moderately Low across all domains. These findings indicate that children referred later showed lower developmental and adaptive functioning at the time of enrollment. Contrary to our hypothesis and to findings reported in studies of older children [18,24], earlier referral in the present sample was not associated with more pronounced clinical impairments. Given the cross-sectional nature of the study, these results do not allow inferences about developmental change over time or the directionality of this association. However, the observed group differences are consistent with evidence from longitudinal studies in infants at elevated likelihood for autism, which have documented increasing divergence in cognitive, adaptive, and behavioral domains between 6 and 24 months of age [68,69]. Although most children in the present sample had not received prior intervention, the potential impact of lacking early developmental support during the first year of life cannot be directly evaluated at this stage and will be addressed in the ongoing longitudinal Telehealth program. Within this framework, the more pronounced clinical impairments observed in children referred later may reflect a more advanced and clinically observable expression of developmental difficulties at the time of enrollment, as increasing developmental demands can amplify existing vulnerabilities. Together, these considerations highlight the importance of monitoring children over time and applying appropriate screening tools (e.g., SACS-R), even when standardized developmental assessments appear adequate.

With regard to the maternal level of experienced stress, 55% of mothers fell within the clinically significant range on the PSI-4 Total Stress scale. This high prevalence is coherent with the broader literature, which consistently shows that parental stress in families of autistic children is markedly elevated relative to reference populations [70,71,72]. Importantly, evidence indicates that clinically significant stress does not arise only in later developmental stages or after prolonged caregiving demands, but is frequently observed already during the preschool years [73]. Higher levels of concern were associated with earlier consultations for early signs of autism, suggesting that some parents are able to detect reduced social orientation and communication during the first year of their infant’s life. This interpretation is supported by recent evidence from Cleary et al. [74], who reported that parents—both in simplex and multiplex families—frequently noted atypical developmental signs as early as 0–12 months, indicating that parental awareness of early social-communication differences often precedes formal diagnosis. It is important to note that infants referred to us before the age of one were not more severely impaired, from a clinical standpoint, than those referred after their first birthday. Indeed, at clinical evaluation they showed similar SACS-R scores to infants enrolled in their second year of life. Furthermore, higher levels of stress on the PSI-4 Child Domain Scale were associated with lower scores on the Vineland-II Communication, Socialization, and Composite scales, indicating that maternal stress was related to core autism features. These findings highlight the importance of attending to parental concerns from an early age, rather than waiting for the full manifestation of symptoms.

### Limitations

A limitation of the current study, although informative in itself, is that the sample may not be representative of the general population, as recruitment was primarily based on self-referral, which was associated with higher parental educational attainment. Consequently, it is possible that families with lower educational or socioeconomic backgrounds may have been less aware of the opportunities to identify autistic prodromes early in infancy and to access support through dedicated and targeted intervention programs. A further consideration is the selection of risk factors, as only a limited set was analyzed and other potentially relevant influences—such as parental age or medication use during pregnancy—were not included, which may have limited the comprehensiveness of the findings. Another potential limitation concerns the unavailability of ADOS-2 data in describing the characteristics of the sample, as this instrument is considered a gold-standard tool for ASD assessment. Administration of the ADOS-2 was not feasible according to the instrument’s criteria, which require a minimum nonverbal developmental age of 12 months and the ability to ambulate independently. Many infants did not meet these criteria because they entered the program during the first year of life. Nevertheless, this limitation was considered acceptable, as the primary objective of the program was to identify infants at increased likelihood of ASD using the validated SACS-R and to evaluate the efficacy of early intervention, with an emphasis on early identification and developmental outcomes rather than definitive diagnostic confirmation.

## 5. Conclusions and Future Directions

In conclusion, these findings demonstrate the possibility of identifying prodromes of autism within the first year of life and highlight the lack of specialized services for very young infants at elevated likelihood for autism in Italy. It is noteworthy that maternal concern emerged as a key factor prompting early consultation. The nationwide distribution of participating families suggests that they were unable to find adequate support in their local areas. Moreover, the fact that the majority of families in our sample contacted us directly, without a referral from their pediatrician, may underscore the need for enhanced pediatric training in early detection for neurodevelopmental disorders, in order to reduce the long-term impact of missed or delayed diagnoses. Furthermore, as self-referral was associated with higher parental educational attainment, broader and more equitable representation across socioeconomic groups may be better achieved through population-based approaches, such as national screening programs embedded within routine pediatric care. In Italy, public healthcare is universally accessible, offering a unique opportunity to reach families across all social strata.

Future studies should investigate the feasibility of longitudinal autism screening (e.g., using the SACS-R) during routine well-child visits and evaluate whether early pediatric referral to neurodevelopmental services for ASD assessment reduces the age at diagnosis and facilitates earlier intervention. Further research is warranted to identify existing gaps in Italian health services regarding early ASD identification and intervention, with the aim of informing and guiding health policies.

## Figures and Tables

**Table 1:** Infants’ Demographic and Clinical Information.

Sample Characterization	Total		Males		Females	
*n*	60		40		20	
Age in months (SD)	10.00 (3.49)		9.84 (3.68)		9.58 (4.00)	
Birth order	30 1st/30 2nd+		22 1st/18 2nd+		8 1st/12 2nd+	
Risk Factors	*n* (%)		*n* (%)		*n* (%)	
ASD Sibling in the Family	13 (21.67%)		7 (17.50%)		6 (30.00%)	
Pregnancy Complications	28 (46.67%)		18 (45.00%)		10 (50.00%)	
Assisted Reproductive Technologies	13 (21.67%)		9 (22.50%)		4 (20.00%)	
Prematurity	9 (15.00%)		5 (12.50%)		4 (20.00%)	
Small for Gestational Age	9 (15.00%)		7 (17.50%)		2 (10.00%)	
No Risk Factor	13 (21.67%)		9 (22.50%)		4 (20.00%)	
Clinical assessment	M (SD)		M (SD)		M (SD)	
Autism-related symptoms						
SACS-R Key Items	4.47	(0.70)	4.50	(0.64)	4.40	(0.88)
Child Development						
Griffiths-III Subscale A	92.61	(15.94)	93.28	(16.69)	91.30	(14.69)
Griffiths-III Subscale B	80.64	(13.92)	83.15	(12.42)	75.75	(15.64)
Griffiths-III Subscale C	88.00	(13.06)	87.31	(13.59)	89.35	(12.19)
Griffiths-III Subscale D	79.19	(14.65)	80.72	(16.13)	76.20	(10.96)
Griffiths-III Subscale E	91.31	(14.39)	90.69	(13.75)	92.50	(15.87)
Griffiths-III General Development	83.17	(15.54)	83.42	(15.72)	82.70	(12.34)
Adaptive Behavior						
Vineland-II Communication Scale	81.42	(8.40)	81.50	(7.65)	81.25	(9.94)
Vineland-II Daily Living Skills Scale	87.95	(9.90)	89.28	(9.22)	85.30	10.88)
Vineland-II Socialization Scale	83.53	(11.85)	84.27	(14.16)	82.05	(13.76)
Vineland-II Motor Skills Scale	89.15	(14.20)	88.95	(14.16)	89.55	(14.63)
Vineland-II Adaptive Composite	83.92	(11.36)	84.30	(10.48)	83.15	(13.20)

Note. 1st = firstborn; 2nd+ = second-born or later.

**Table 2:** Overview of Recruitment Pathways and Parental Concerns.

		Medical Referral *n* (%)	Self-Referral *n* (%)
		23 (38%)	37 (62%)
Parental Concern (DSM-5 Categories)	A + B	10 (43.48%)	16 (43.24%)
	A	11 (47.83%)	20 (54.05%)
	B	0 (0%)	1 (2.70%)
	Other	0 (0%)	0 (0%)
	None	2 (8.70%)	0 (0%)

Note: Category A = Concerns reported by parents only in the area of social communication and social interaction; Category B = Concerns reported by parents only in the area of restricted, repetitive patterns of behavior, interests, or activities. A + B = Concerns reported by parents in both areas.

**Table 3:** Parents’ Demographic Information.

**Age (Years)**	**Mothers**	**Fathers**
M (SD)	34.75 (4.84)	37.40 (5.58)
Range	27–45	22–44
**Educational level**	*n* (%)	*n* (%)
Doctoral degree	2 (3.39%)	3 (5.00%)
University degree	34 (57.63%)	19 (31.67%)
High school diploma	19 (32.20%)	23 (38.33%)
Middle School diploma	4 (6.68%)	15 (25.00%)

**Table 4:** Family Educational Attainment by Type of Referral.

Family Educational Attainment	Self-Referral	Medical Referral	Total
Higher Education	31 (84%)	9 (39%)	40 (67%)
Lower Education	6 (16%)	14 (61%)	20 (33%)
Total	37	23	60

Note. Values are presented as *n* (column %). Pearson χ^2^(1, *n* = 60) = 12.73, *p* < 0.001; Continuity Correction χ^2^(1) = 10.80, *p* = 0.001; Fisher’s Exact Test *p* < 0.001.

**Table 5:** Presence of Risk Factors by Age at Enrollment.

Risk Factors	Age < 12 Months	Age ≥ 12 Months	Total
At least one risk factor	32 (76%)	15 (83%)	47 (78%)
No risk factor	10 (24%)	3 (17%)	13 (22%)
Total	42	18	60

Note. Values are presented as *n* (column %). Chi-square test indicated no significant association between variables, χ^2^(1, *n* = 60) = 0.38, *p* = 0.54; Fisher’s Exact Test confirmed the absence of a significant association (*p* = 0.74).

**Table 6:** Comparison between the Clinical Profiles of Infants who were referred to our Service <12 m vs. ≥12 m.

	Infants < 12 m (*n* = 42)		Infants ≥ 12 m (*n* = 18)		Student’s *t*-Test				
	M	(SD)	M	(SD)	t	*p* Value	Cohen’s d	95% CI	Magnitude
Griffiths III									
Foundations of Learning	98.02	(13.79)	80.27	(14.09)	4.50	<0.001 ***	1.28	[0.67, 1.88]	Large
Language and Communication	83.92	(13.04)	73.00	(13.45)	2.92	<0.01 **	0.83	[0.25, 1.40]	Large
Eye and Hand Coordination	92.05	(9.97)	79.05	(15.18)	3.32	<0.01 **	1.10	[0.51, 1.69]	Large
Personal-Social-Emotional	84.47	(11.09)	67.67	(15.56)	4.69	<0.001 ***	1.33	[0.72, 1.94]	Large
Gross Motor	96.00	(10.05)	80.94	(17.58)	3.39	<0.01 **	1.18	[0.57, 1.77]	Large
General Development	89.87	(8.97)	68.50	(14.03)	6.95	<0.001 ***	1.98	[1.30, 2.64]	Very large
Vineland-II									
Communication	84.12	(7.26)	75.33	(7.98)	4.15	<0.001 ***	1.17	[0.58, 1.76]	Large
Daily Living Skills	89.51	(10.50)	84.28	(7.74)	1.89	0.06	0.54	[−0.03, 1.10]	Medium
Socialization	86.07	(10.95)	78.22	(12.47)	2.43	0.02 *	0.69	[0.12, 1.25]	Medium
Motor Skills	93.60	(13.12)	78.50	(11.03)	4.26	<0.001 ***	1.21	[0.60, 1.80]	Large
Adaptive Behavior Composite	87.85	(10.02)	75.00	(9.49)	4.60	<0.001 ***	1.30	[0.70, 1.90]	Large

* *p* < 0.05; *p* < 0.01 **; *p* < 0.001 ***. Note. Cohen’s d represents standardized mean differences between groups, with 95% confidence intervals (CI). Effect size magnitude was interpreted using conventional benchmarks (small = 0.20, medium = 0.50, large = 0.80). Values ≥ 1.50 were additionally described as very large to facilitate interpretation of effects of substantial magnitude.

## Data Availability

The data presented in this study are not publicly available due to privacy restrictions; however, they are available upon request from the corresponding author.

## Abbreviations

The following abbreviations are used in this manuscript:

ASD	Autism Spectrum Disorder
ART	Assisted Reproductive Technologies
DQ	Developmental Quotient
PSI-4	Parenting Stress Index—Fourth Edition
RCT	Randomized Controlled Trial
SACS-R	Social Attention and Communication Surveillance-Revised
SGA	Small for Gestational Age
